# MicroRNA-23a promotes neuroblastoma cell metastasis by targeting CDH1

**DOI:** 10.3892/ol.2014.1794

**Published:** 2014-01-14

**Authors:** LIN CHENG, TAO YANG, YONGQIN KUANG, BIN KONG, SIXUN YU, HAIFENG SHU, HUTIAN ZHOU, JIANWEN GU

**Affiliations:** Department of Neurosurgery, Chengdu Military General Hospital, Chengdu, Sichuan 610083, P.R. China

**Keywords:** miRNA, miR-23a, CDH1, neuroblastoma

## Abstract

CDH1 inactivation is important in tumor metastasis. In the present study, it was suggested that the mRNA and protein levels of CDH1 decreased in metastatic neuroblastoma (NB) tissues compared with those in primary NB tissues. The aim of the study was to explore the regulatory mechanisms of CDH1 downregulation in metastatic NB. MicroRNAs are small non-coding RNAs (~22 nt in length) that negatively regulate target mRNAs and are involved in various cancer-related processes, including metastasis. In the current study, miR-23a was shown to be upregulated in human metastatic NB tissues compared with primary NB tissues. Inhibition of miR-23a may significantly suppress NB cell migration and invasion. *In vitro* reporter assay suggested that CDH1 is a direct target gene of miR-23a. Furthermore, blocking the expression of miR-23a partly restored the expression of CDH1 in NB cells. These findings provide evidence that miR-23a is key in promoting NB cell migration and invasion through targeting CDH1, and suggest that exogenous miR-23a may have therapeutic value in treating NB metastasis.

## Introduction

Characterized by spontaneous regression, maturation or aggressive progression, neuroblastoma (NB) is one of the most common forms of cancer in young children. NB typically presents with metastatic disease at diagnosis with a poor outcome (1,2). The most critical step in NB metastasis is that the cells from the primary tumor, which have enhanced capabilities of migration and invasion, invade into the surrounding normal tissues (3). To date, a set of genes associated with tumor progression in NB have been revealed (4). However, the precise mechanisms, particularly the significant molecular pathways in the transition from primary to metastatic NB, remain to be completely understood.

The *CDH1* gene encodes the epithelial cell adhesion molecule, E-cadherin, which forms the core of the adherens junctions between adjacent epithelial cells (5). The significance of CDH1 inactivation for metastasis has been demonstrated in a variety of studies (6–11). However, few studies have analyzed the potential important roles of CDH1 in metastatic NB tissues.

Previously, a group of non-coding single-stranded RNAs, ~22 nt in length, which modulate gene expression post-transcriptionally by interacting with complementary sites within the 3′ untranslated region (UTR) of target mRNA, have emerged as important regulators in cancer-related processes, including metastasis (12–15). In a previous study, Guo *et al* observed differential expression of microRNAs (miRNAs) in metastatic NB compared with primary NB using microarray analysis. In addition, the potential roles of these miRNAs in the NB metastatic process were investigated (16). However, the role of miR-23a in metastatic NB tissues compared with primary NB tissues remains unclear. Hence, we proposed that the upregulated miR 23a enhances the metastatic capability of NB cells. Exploring the miR-23a-mediated NB metastasis molecular mechanism may be useful to characterize the progression from primary to metastatic NB.

## Materials and methods

### Tissue samples and cell lines

In total, nine pairs of human primary and metastatic NB tissues were acquired from the Chengdu Military General Hospital (Chengdu, China). The study was approved by the ethics committee of Chengdu Military General Hospital and informed written consent was obtained from the patients. All the tissues were confirmed by pathology and immunohistochemistry, frozen in liquid nitrogen and stored at −80°C. Human NB cell lines, SK-N-SH and GI-LA-N, were cultured in Eagle’s minimum essential medium (Gibco-BRL, Carlsbad, CA, USA) supplemented with 10% FBS and 100 U/ml penicillin/streptomycin. All the cells were placed in a humidified incubator with 5% CO_2_ at 37°C.

### RNA isolation and qPCR

Total RNA was isolated using TRIzol reagent (Invitrogen Life Technologies, Carlsbad, CA, USA) according to the manufacturer’s instructions. The RNA concentration and quality was measured by a NanoDrop ND-1000 spectrophotometer (Thermo Fisher Scientific, Waltham, MA, USA). Next, 1 μg RNA was used for reverse transcription (RT). Specific miR-23a reverse transcription (RT) primers were used for the cDNA from the miRNA RT reaction, while the common primer Oligo (dT) was used for the cDNA from total RNA, for detection of CDH1. qPCR was performed using the SYBR Green PCR master mix (Applied Biosystems, Inc., Foster City, CA, USA) according to the following conditions: 95°C for 5 min followed by 40 cycles of amplification at 95°C for 30 sec, 57°C for 30 sec and 72°C for 30 sec. U6 small nuclear B non-coding RNA was used as the internal control to normalize miR-23a expression and GAPDH was used as the internal control to normalize CDH1 expression.

### Western blot analysis

The NB cells were seeded into six-well plates at a density of 3×10^4^ cells/well and cells were transfected once cell density reached ~80% confluence on the second day. At 48 h following transfection, the cells were lysed using RIPA buffer for 30 min at 4°C. The protein concentration was measured by the bicinchoninic acid method using a Pierce^®^ BCA Protein Assay kit (Thermo Fisher Scientific) and then 20 μg protein was loaded on SDS-PAGE for analysis. The primary antibodies used were rabbit polyclonal anti-human CDH1 (1:500; Abcam, Cambridge, MA, USA) and rabbit monoclonal anti-human GAPDH (1:1,000; Abcam). The secondary antibody used was a goat anti-rabbit IgG conjugated with horseradish peroxidase (1:1,000; Abcam). The bound antibodies were detected by ECL Plus Western blotting detection system (GE Healthcare, Amersham, UK) and the chemiluminescent signals were detected by high-performance chemiluminescence film (GE Healthcare).

### EGFP reporter assay

The 3′UTR of CDH1 was amplified and cloned downstream of the pcDNA3/EGFP vector. Next, the mutant 3′UTR of CDH1 (in which several nucleotides within the binding sites were deleted) was amplified using pcDNA3/EGFP-CDH1 3′UTR as the template and cloned downstream of the pcDNA3/EGFP vector. For the EGFP reporter assay, the cells were co-transfected with miR-23a mimics, ASO-miR-23a and pcDNA3/EGFP-CDH1 3′UTR or the mutant 3′UTR, together with the controls. The plasmid expressing RFP was transfected as the spike-in control. At 48 h following transfection, the cells were lysed using RIPA buffer, and EGFP and RFP intensity was measured by an F-4500 fluorescence spectrophotometer (Hitachi, Ltd., Tokyo, Japan).

### Transwell migration and invasion assays

The migration and invasion assays were performed using the Transwell chamber (Millipore, Billerica, MA, USA). For the migration assay, following transfection, the cells were seeded into the upper chamber (2.5×10^4^ cells/well) in 250 μl serum-free medium, but the bottom chamber was incubated with 750 μl medium containing 10 or 20% serum. For the invasion assay, the chamber was coated with Matrigel and the cells were seeded into the upper chamber (2.5×10^4^ cells/well) in 250 μl serum-free medium, while the bottom chamber was incubated with 750 μl medium containing 10 or 20% serum. Following cell migration or invasion for 20 h, the cells that had not migrated or invaded were scraped off with a cotton swab. The cells which had migrated or invaded into the chamber membrane were fixed and then stained with crystal violet for ~15 min. Finally, images of the cells were captured under a microscope (ME21 digital microscope, Olympus, Guangzhou, China) and cells were counted.

### Statistics

Data are presented as the mean ± standard deviation and the difference between groups was determined by a two-tailed Student’s t-test. P<0.01 or P<0.05 were considered to indicate a statistically significant difference.

## Results

### Quantitative analysis of CDH1 expression in metastatic NB tissues

**Figure 1:**
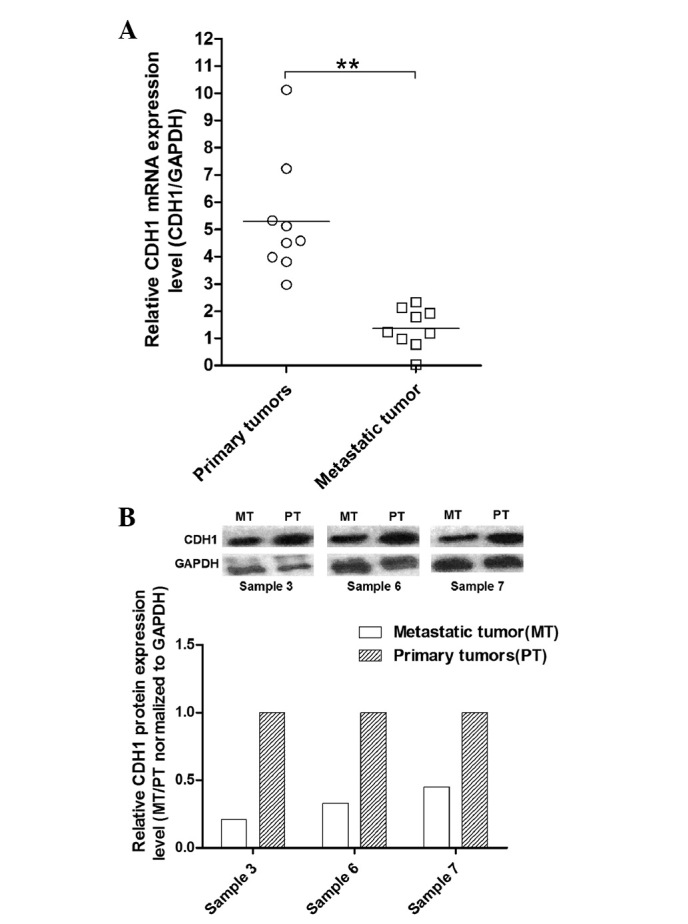
CDH1 is downregulated in metastatic NB tissues compared with primary NB tissues. The expression levels of CDH1 (A) mRNA and (B) protein were analyzed by real-time polymerase chain reaction and western blot analysis, respectively and were normalized to GAPDH in metastatic and primary NB tissues. The representative results of patients 3, 6 and 7 are shown. ^**^P<0.01. NB, neuroblastoma.

CDH1 is an important cancer metastasis-related molecule. To test the expression of CDH1 in metastatic NB tissues and matched primary NB tissues, qPCR assay was performed in nine pairs of NB tissues. It was shown that CDH1 mRNA expression levels were generally and significantly (P<0.01) lower in metastatic NB tissues than in the matched primary NB tissues (Fig. 1A). In addition, western blot analysis confirmed that the CDH1 protein expression was lower in metastatic NB tissues compared with that in primary NB tissues within the representative patients 3, 6 and 7.

### miR-23a is a candidate regulator of CDH1 in NB

**Figure 2:**
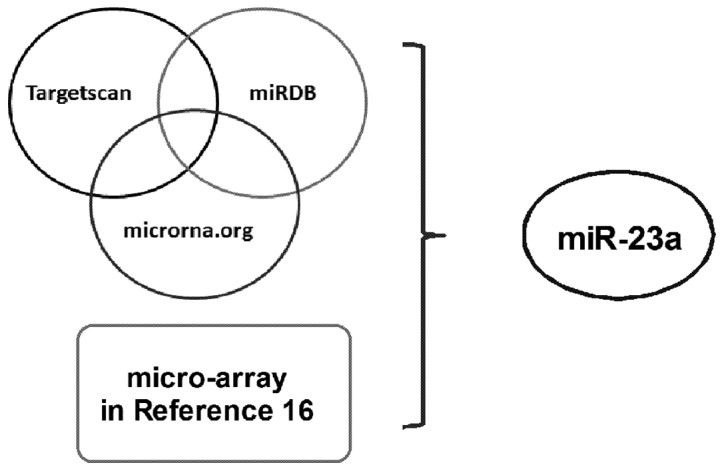
miR-23a is a candidate regulator of CDH1 in NB. miR-23a simultaneously appeared among the 20 miRNAs shown in Table I (miRNAs that may potentially target the 3′ untranslated region of CDH1, predicted by TargetScan, microRNA.org and miRDB) and 34 miRNAs shown in Table II, which were upregulated in metastatic NB tissues compared with primary NB tissues. NB, neuroblastoma.

miRNAs function as tumor suppressors or oncogenes, through the direct regulation of associated oncogenes or tumor suppressor genes (14,17,18). The oncogene miRNAs (onco-miRs), such as miR-23a, are usually upregulated in tumors and may cause the loss of expression of tumor suppressors and contribute to the tumorigenesis of cancer, including metastasis. The loss of CDH1 expression in metastatic NB tissues implies that there may be important miRNAs involved in the regulation of CDH1 in NB metastasis. Greater specificity in miRNA predictions may be attained by the consensus of multiple algorithms. Therefore, three programs (TargetScan, microRNA.org and miRDB) were used for the prediction of which miRNAs target the 3′UTR of CDH1 and regulate its expression. Table I shows the 20 miRNAs that were identified to target the 3′UTR of CDH1. In addition, Table II shows the 34 downregulated miRNAs collected from metastatic NB tissues to compare with primary NB tissues, according to the previous study by Guo *et al* using the microarray analysis (16). Based on these two tables, miR-23a (Fig. 2) was identified as a candidate for directly targeting CDH1 whose mRNA 3′UTR contains a putative binding site. This analysis is consistent with the model in which tumor onco-miRs were found to promote tumor development by targeting and negatively regulating tumor suppressors (13).

### miRNA-23a is upregulated in metastatic NB tissues and regulates NB cell migration and invasion

**Figure 3:**
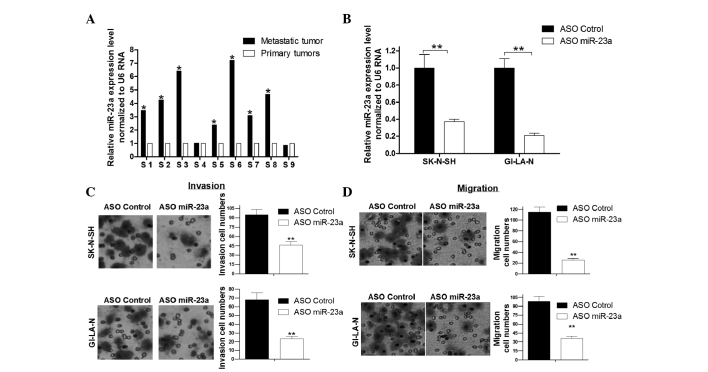
miR-23a is upregulated in metastatic NB tissues and suppresses the NB cell metastasis. (A) qPCR was performed to test the expression of miR-23a in the nine pairs of metastatic and primary NB tissues and was normalized to U6. (B) The SK-N-SH and GI-LA-N cells were transfected with ASO miR-23a, together with the controls. The miR-23a expression levels were measured by qPCR and normalized to U6. (C) Invasion and (D) migration assays were performed to detect the effects of miR-23a on the invasion and migration capabilities of SK-N-SH and GI-LA-N cells. The NB cells were seeded onto Transwell chambers for migration (without Matrigel) and invasion (with Matrigel). These assays were performed three times for each group; the number of migratory or invasive cells per field (graphs) and the invasive and migratory SK-N-SH and GI-LA-N cells stained with crystal violet (images) are presented. ^**^P<0.01. NB, neuroblastoma; qPCR, real-time polymerase chain reaction; UTR, untranslated region; miRNA, microRNA.

miR-23a has been previously reported to play a role as an onco-miR in several types of cancer, and the microarray analysis identified that miR-23a is upregulated in metastatic NB tissues compared with primary NB tissues. Next, qPCR assay was used to detect the expression of miR-23a in nine pairs of NB samples (metastatic and matched primary NB tissues). It was shown that miR-23a expression levels were generally higher in metastatic NB tissues compared with primary NB tissues (Fig. 3A). Transwell migration (without Matrigel) and invasion (with Matrigel) assays were used to evaluate the effect of miR-23a on NB cell migration and invasion. As Fig. 3C shows, blocking the expression of miR-23a with miR-23a ASO significantly decreased the number of invaded SK-N-SH and GI-LA-N cells compared with the ASO control group. This implies that the upregulated miR-23a promotes the invasion of NB cells. Consistent with the invasion assay results, blockage of miR-23a significantly inhibited the migration of SK-N-SH and GI-LA-N cells compared with the control group (Fig. 3D). Thus, it was concluded that miR-23a is overexpressed in metastatic NB tissues and inhibition of miR-23a suppresses the migration and invasion of NB cells.

### miR-23a directly targets CDH1 expression in NB cells

**Figure 4:**
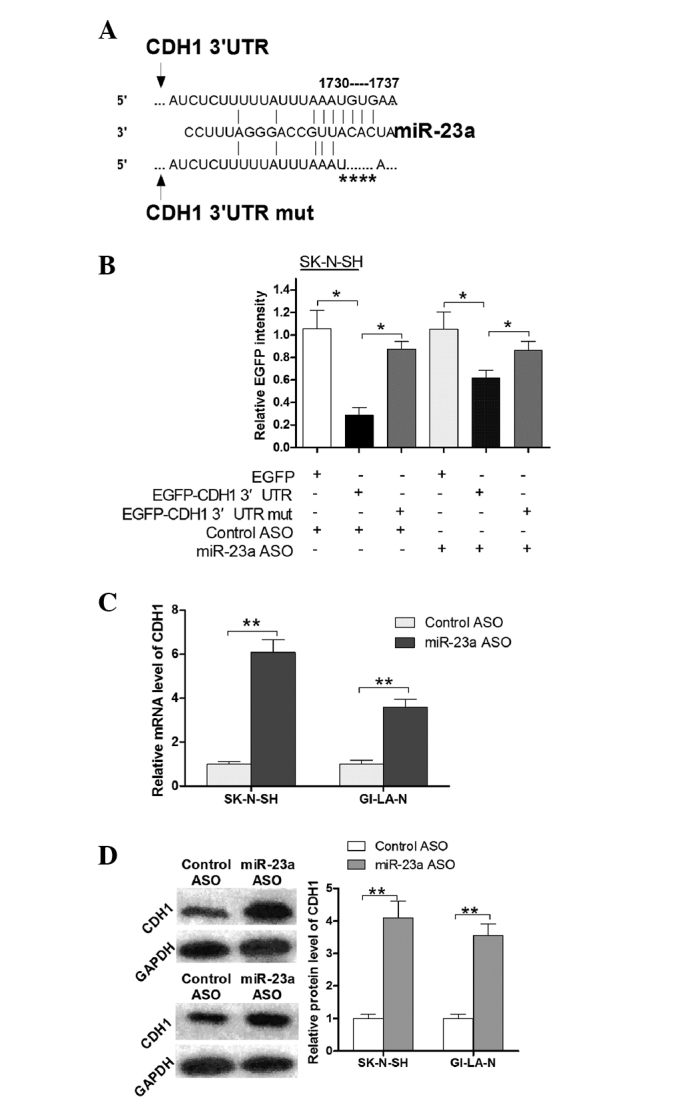
CDH1 is a direct target of miR-23a. (A) Algorithms between miR-23a and the 3′UTR and mutant 3′UTR of CDH1 (in which several nucleotides within the binding sites were deleted). (B) EGFP reporter assay was performed to detect the effect of miR-23a on the EGFP intensity controlled by the 3′UTR of potential targets. (C) qPCR and (D) western blot analysis were performed to detect the effect of miR-23a on CDH1 mRNA and protein expression, respectively, in SK-N-SH and GI-LA-N cells. ^**^P<0.01. UTR, untranslated region; qPCR, real-time polymerase chain reaction.

To verify that the regulation of CDH1 expression by miR-23a is direct, an EGFP reporter system was used in SK-N-SH cells. The alignment of miR-23a with the CDH1 3′UTR insert is illustrated in Fig. 4A. In addition, a mutated 3′UTR was constructed by deleting 4 nt in the seed sequence of miR-23a (asterisk marked in Fig. 4A). The EGFP reporter analysis showed that with the CDH1-3′UTR insert, the EGFP intensity was significantly lower than that in the CDH1-3′UTR-mutant group when treated with control oligo. When miR-23a was blocked by miR-23a ASO, the EGFP intensity levels were significantly higher than those in the control group. However, the EGFP intensity with the mutated 3′UTR was not affected by miR-23a (Fig. 4B). Next, it was investigated whether the endogenous CDH1 is regulated by miR-23a in NB cells. qPCR assay revealed that the CDH1 mRNA levels were inversely correlated with the miR-23a levels (Fig. 4C). The trend of CDH1 protein levels was similar to that of the mRNA levels, according to the western blot analysis (Fig. 4D). Therefore, it was concluded that CDH1 is a direct target of miR-23a in SK-N-SH cells, and that miR-23a regulates the expression of CDH1 at the mRNA and protein levels.

### CDH1 suppresses NB cell migration and invasion in vitro

**Figure 5:**
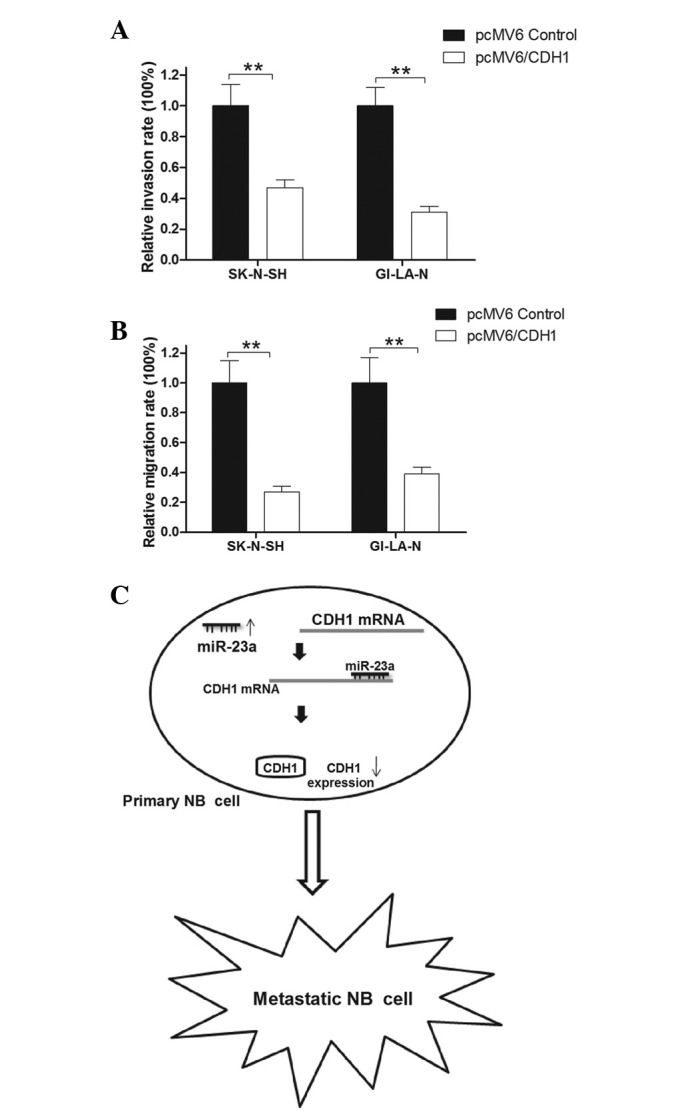
CDH1 suppresses NB cell migration and invasion *in vitro.* SK-N-SH and GI-LA-N cell (A) invasion and (B) migration capabilities were investigated by Transwell assay. The cells were transfected with pcMV6/CDH1 or control and the rate of migration and invasion were measured by the cell number of migrated and invaded cells, respectively, in the CDH1 group compared with the control group. (C) Schematic representation showing that the upregulated miR-23a in primary NB cells directly causes the downregulation of CDH1 and promotes the transformation of primary to metastatic NB cells. NB, neuroblastoma.

Previous studies have shown that the downregulation of CDH1 is an important feature of a number of transformed cells (19), and that CDH1 functions as a tumor suppressor. Accordingly, the present study investigated whether CDH1 affects NB cell metastasis. Overexpression plasmids (pCMV6/CDH1) were constructed that ectopically overexpress the CDH1 protein in NB cells. Subsequently, the effect of CDH1 on cell metastasis was evaluated by Transwell assay. The results showed that the invasion rate was reduced by ~50 and ~60% with the overexpression of CDH1 in SK-N-SH and GI-LA-N cells, respectively, compared with that of the control group (Fig. 5A). In addition, the migration rate of the SK-N-SH and GI-LA-N cells transfected with pCMV6/CDH1 was markedly lower than that of the control group (Fig. 5B). These results provided further evidence that CDH1 is a tumor suppressor in NB metastasis. The tumor suppressor role of CDH1 in NB metastasis may explain the manner in which the upregulation of miR-23a promotes NB cell migration and invasion and contribute to the transformation of primary to metastatic NB (Fig. 5C).

## Discussion

As in the case of various types of cancer metastasis, the transformation of primary to metastatic NB is a complex multistep process including cell adhesion, migration, angiogenesis, immune escape and homing to target organs. The increased NB cell migration and invasion capabilities are essential features of the metastatic transformation process. Identifying the molecules and pathways that control NB cell migration and invasion is critical to understanding NB metastasis.

Accumulating previous evidence has suggested that genetic or epigenetic alterations in CDH1 or alterations in their protein expression, often result in tissue disorder, cellular dedifferentiation, increased invasiveness of tumor cells and ultimately metastasis (19–21). It has been previously demonstrated that the downregulation of CDH1 leads to loss of cell polarity and derangement of normal tissue architecture (22,23). In various types of cancer, CDH1-mediated cell-cell adhesion is lost concomitantly with acquisition of an invasive phenotype, high tumor grade and low patient survival rates (24–29). Based on the important role of CDH1 in cancer tumorigenesis, the investigation of the regulatory mechanisms of CDH1 in the process of primary to metastatic NB transition may highlight new prognostic markers and therapy targets for the metastasis of NB in the future. To date, somatic mutations, chromosomal deletions, proteolytic cleavage and silencing of the CDH1 promoter have been reported to cause the loss or reduction of CDH1 expression (30–32). In a previous study by Hoebeeck *et al*, the authors tested 42 primary NB tumors and the frequencies of methylation were 8% with CDH1 (19). This implies that there may be potential other mechanisms that are involved in the downregulation of CDH1 in the NB metastatic process. In the present study, nine pairs of metastatic NB tissues and matched primary NB tissues were tested, and the mRNA and protein levels of CDH1 were found to be significantly decreased in the NB metastatic transformation process (Fig. 1).

Previously, miRNAs have been identified as important regulators of gene expression. Several miRNAs have been found to exhibit prometastatic (miR-10b, -21 and -373/520c) or antimetastatic (miR-34b/c, -126, -148a, -206 and -335) activity (33–35). In a previous study, miRNA microarray assay was performed to screen out the miRNAs that were differentially expressed between metastatic and primary NB tissues (16). In addition, a previous study has shown that miR-9, which is directly bound and upregulated by MYC and MYCN in breast cancer cells, directly targets CDH1, leading to increased cell motility and invasiveness (26). miR-10b has been found to modulate breast cancer metastasis by targeting CDH1 and may be a useful biomarker of advanced progression and metastasis of breast cancer (36). miR-25 has also been demonstrated to promote esophageal squamous cell carcinoma cell migration and invasion by directly targeting CDH1 (37). Recently, Cao *et al* (38) found that miR-23a regulates TGF-β-induced epithelial-mesenchymal transition by targeting E-cadherin (CDH1) in lung cancer cells. However, few studies have considered the manner in which the miRNAs contribute to the transformation of primary to metastatic NB through the CDH1-induced pathway. Therefore, the current study analyzed the microarray results (Table II) and miRNA target prediction algorithms (Table I). miR-23a was identified as a candidate molecule in the regulation of NB metastasis by the CDH1 pathway (Fig. 2).

miR-23a belongs to the miR-23a/24/27a cluster, which is located in chromosome 19p13.12 and may be induced by TGF-β. In several types of human cancer, this gene cluster functions as an oncogenic miRNA and has been previously reported to be upregulated in types of human cancer. As one of the most famous members of the miRNA-cluster, miR-23a has been shown to promote the growth of gastric adenocarcinoma cells by targeting the interleukin-6 receptor (39,40). miR-23a has also been demonstrated to promote colon carcinoma cell growth, invasion and metastasis through inhibition of the MTSS gene (41,42). The current study revealed that miR-23a is upregulated in metastatic NB tissues when compared with primary NB tissues. In addition, blockage of miR-23a may reduce the capability of NB cell migration and invasion using Transwell assays (Fig. 3). To identify the direct regulation of CDH1 by miR-23a in NB cells, qPCR, western blot analysis and EGFP report system were used to demonstrate that CDH1 is a direct target gene of miR-23a (Fig. 4). The Transwell assay results by overexpression of CDH1 in SK-N-SH and GI-LA-N cells showed that the CDH1 may inhibit the capability of NB cell migration and invasion (Fig. 5). This was found to correlate with the blocking of the expression of miR-23a. These results suggest that there exists a miR-23a/CDH1 pathway, which is important in the regulation of NB, particularly in the process of primary to metastatic NB transition (Fig. 5C).

In conclusion, the results of the present study may be summarized by the following six major observations: i) CDH1 was inactive in the metastatic NB tissues compared with the primary NB tissues at the mRNA and protein levels; ii) bioinformatic software, including TargetScan, microRNA.org and miRDB algorithms, were used and miR-23a was identified as a candidate regulator of CDH1 in the transformation process of primary to metastatic NB; iii) miR-23a was found to be upregulated in metastatic NB tissues compared with primary NB tissues; iv) CDH1 is negatively regulated by miR-23a at the mRNA and protein levels and is a direct target in NB; v) inhibition of miR-23a suppresses the migration and invasiveness of SK-N-SH and GI-LA-N cells; and vi) there exists a miR-23a/CDH1 pathway in the transition of primary to metastatic NB. However, whether miR-23a may become a new prognostic marker and therapy target for the metastasis of NB must be explored in future studies.

## Figures and Tables

**Table I:** Predicted miRNAs targeting CDH1 by three algorithms.

Gene name	miRNA	microRNA.org	miRDB	TargetScan
CDH1	hsa-miR-1248	+	+	+
CDH1	hsa-miR-219-2-3p	+	+	+
CDH1	hsa-miR-504	+	+	+
CDH1	hsa-miR-340	+	+	+
CDH1	hsa-miR-580	+	+	+
CDH1	hsa-miR-296-3p	+	+	+
CDH1	hsa-miR-1299	+	+	+
CDH1	hsa-miR-1296	+	+	+
CDH1	hsa-miR-515-5p	+	+	+
CDH1	hsa-miR-1323	+	+	+
CDH1	hsa-miR-1264	+	+	+
CDH1	hsa-miR-548o	+	+	+
CDH1	hsa-miR-619	+	+	+
CDH1	hsa-miR-544	+	+	+
CDH1	hsa-miR-339-5p	+	+	+
CDH1	hsa-miR-23b	+	+	+
CDH1	hsa-miR-23a	+	+	+
CDH1	hsa-miR-1207-5p	+	+	+
CDH1	hsa-miR-219-5p	+	+	+
CDH1	hsa-miR-338-3p	+	+	+

miRNA, microRNA.

**Table II:** Differential expression of miRNAs in NB between metastatic and primary tumors (increased expression, >2-fold).

miRNA	Fold changes (metastatic/primary tumors)
hsa-miR-342-3p	6.2879
hsa-miR-92b	11.8088
hsa-miR-130b	2.5771
hsa-miR-129-3p	3.0205
hsa-miR-483-3p	9.9583
hsa-miR-345	3.1005
hsa-miR-24	5.0395
hsa-miR-486-5p	2.4739
hsa-miR-99b	3.2086
hsa-miR-145	4.9442
hsa-miR-375	3.5245
hsa-miR-92a	5.3560
hsa-miR-361-5p	4.0653
hsa-miR-484	2.9674
hsa-miR-339-5p	3.8349
hsa-miR-148b	4.3914
hsa-miR-140-3p	3.7209
hsa-miR-335	2.5920
hsa-miR-483-5p	3.2567
hsa-miR-23a	2.5497
hsa-miR-29a	2.4728
hsa-miR-423-3p	2.0051
hsa-miR-128a	2.3610
hsa-miR-130a	3.1655
hsa-miR-214	2.8887
hsa-miR-15a	2.5875
hsa-miR-191	2.3789
hsa-miR-100	2.2647
hsa-miR-10b	3.1618
hsa-miR-29b	2.4477
hsa-miR-30c	2.5702
hsa-miR-324-5p	2.2561
hsa-miR-602	4.4085
hsa-miR-107	2.1387

Taken from (16). miRNA, microRNA; NB, neuroblastoma.
